# Understanding Components of Therapeutic Alliance and Well-Being from Use of a Global Digital Mental Health Benefit During the COVID-19 Pandemic: Longitudinal Observational Study

**DOI:** 10.1007/s41347-022-00263-5

**Published:** 2022-07-13

**Authors:** Sara J. Sagui-Henson, Camille E. Welcome Chamberlain, Brooke J. Smith, Elizabeth J. Li, Cynthia Castro Sweet, Myra Altman

**Affiliations:** 1 Modern Health, San Francisco, CA USA; 2 Clinical Excellence Research Center, Stanford School of Medicine, Palo Alto, CA USA

**Keywords:** Digital mental health, Therapeutic alliance, Teletherapy, Telecoaching, Well-being, COVID-19, Videoconferencing

## Abstract

Digital mental health services leverage technology to increase access to care, yet less is known about the quality of therapeutic relationships in a virtual setting. This study examined components of therapeutic alliance (a mechanism underlying successful treatment) and its association with beneficial treatment outcomes in a real-world, virtual setting. The objective is to examine (1) participant ratings of components of therapeutic alliance with providers in a virtual setting, (2) changes in subjective well-being and depressive symptoms among participants who began care with elevated depressive symptoms, and (3) the association between components of alliance and changes in participants’ well-being. Adults (*N* = 3,087, *M* age = 36 ± 9 years, 54% female) across the world with access to digital mental health benefits who engaged in videoconference sessions with a licensed therapist (18%, 555/3,087), certified coach (65%, 2,003/3,087), or both (17%, 529/3,087) between Sept. 29, 2020 and Oct. 12, 21. Participants completed 2 adapted items from the Working Alliance Inventory (goals and bonds subscales) after each session, and ratings were averaged across visits (Cronbach’s *ɑ* = .72). Participants’ World Health Organization-Five (WHO-5) Well-Being Index scores at the start and end of the study period were used to measure changes in subjective well-being. Descriptive and inferential statistics were conducted to examine average alliance ratings across demographics and utilization types and the association between alliance and well-being. The median adapted therapeutic alliance score was 4.8 (range: 1–5) and did not differ by age, country, or baseline well-being (*P*s > .07). Females reported higher components of alliance than males (4.88 vs. 4.67, *P* = .01). Participants utilizing telecoaching reported higher components of alliance than those utilizing teletherapy or both telecoaching and teletherapy (4.83 v. 4.75, *P* = .004), though effect sizes were negligible. Among those with elevated baseline depressive symptoms (*n* = 835), participants reported an average WHO-5 increase of 15.42 points (95% CI 14.19–16.65, *P* < .001, Cohen *d* = 1.06) with 58% (485/835) reporting clinical recovery and 57% (481/835) reporting clinical improvement in depressive symptoms. Higher components of therapeutic alliance scores predicted greater well-being at follow-up (*b* = 2.04, 95% CI 0.09–3.99, *P* = .04) after controlling for age, sex, baseline WHO-5, and number of days in care (*R*^*2*^ = .06, *P* < .001). Exploratory analyses indicated this association did not differ by utilization type, baseline well-being, or session utilization (*P*s > .34). People with access to one-on-one videoconferencing care via a digital mental health benefit formed a strong bond and sense of alignment on goals with both coaches and therapists. Higher components of alliance scores were associated with improvements in subjective well-being among participants who began care with elevated depressive symptoms, providing evidence that a positive bond and goal alignment with a provider are two of many factors influencing virtual care outcomes. Continued focus on the quality of therapeutic relationships will ensure digital mental health services are patient-tailored as these platforms expand equitable access to evidence-based care.

## Introduction

Since the onset of the COVID-19 pandemic, the importance of timely and equitable access to high-quality, evidence-based, and outcome-driven mental health treatment has reached an all-time high (Kumar & Nayar, 2021; Wind et al., 2020). The prevalence of mental health issues, including depression, anxiety, and general mental distress, has increased dramatically (Wu et al., 2021), particularly for individuals with a prior history of mental health issues (Sherman et al., 2020). Given the physical distancing measures and government-mandated lockdowns put in place to limit the spread of COVID-19, utilization of digital mental health services increased significantly to promote continuity and equitable access to care (Mosnaim et al., 2020). The types of mental health services offered through digital means are comprehensive, with most one-on-one care shifting to videoconferencing meetings or phone calls. Although telehealth services produce similar outcomes to in-person treatment (Krzyzaniak et al., 2021; Thomas et al., 2021), questions remain about whether a virtual setting is conducive to the development and maintenance of a strong therapeutic alliance between patients and providers (Simpson & Reid, 2014).

Therapeutic alliance, also referred to as the working alliance, the helping alliance, or simply the alliance, refers to the quality and collaborative aspects of the patient-provider relationship that may underlie the effectiveness of different therapeutic practices (Flückiger et al., 2018; Horvath et al., 2011). The alliance is characterized by several relationship qualities and facilitative conditions that help patients grow and heal in mental health treatment. These can include acceptance, empathetic understanding, openness, and a collaborative partnership (Agnew-Davies et al., 1998). In the original conceptualization of therapeutic alliance, Bordin (1979) specifically outlined three components that are important to the development of a strong relationship: agreement on therapeutic goals, agreement on therapeutic tasks, and a positive bond.

Agreement on therapeutic goals refers to the collaborative identification of the patient’s treatment goals that capture their primary struggles and match their presenting problem (Bordin, 1994). Agreement on therapeutic tasks refers to specific activities that patients will engage in to facilitate change and progress toward their goals. These should be set collaboratively and involve the tasks the partnership agrees need to be enacted in the service of positive change (Bordin, 1994). A positive bond covers several aspects of the interpersonal relationship between patients and providers. There are many ways to build this bond, such as listening empathetically, attending to boundaries, and encouraging mutual respect, which result in the patient feeling trust and confidence that the work with their provider will help them achieve their goals (Bordin, 1994). Some research on alliance in cognitive behavioral therapy suggests that the goal and task components represent one factor that is independent of the bonded relationship factor (Andrusyna et al., 2001). Therapeutic alliance is dynamic and may evolve over time as the patient and provider develop a shared sense of understanding and commitment to the patient’s goals (Luborsky, 1976).

Therapeutic alliance is a core mechanism for change and the most reliable predictor for both treatment outcomes and attrition (Horvath et al., 2011; Wampold & Imel, 2015). In in-person therapy settings, stronger alliance is linked to better mental health outcomes, such as improved psychological well-being (Alessi et al., 2019), decreased depression symptomatology (Arnow et al., 2013; Cameron et al., 2018; Klein et al., 2003; Laws et al., 2017), and decreased social anxiety symptomatology (Kivity et al., 2021). However, less is known about therapeutic alliance in videoconferencing settings, where the development of a strong bond may unfold differently when patients and providers are not physically in the same space.

The use of videoconferencing for delivering mental health treatment presents some challenges and opens new possibilities for creating a strong alliance. Some clinicians report difficulty judging non-verbal behavior (Thomas et al., 2021) and hold a belief that technological disruptions may be a barrier to developing rapport (Cowan et al., 2019; Morland et al., 2010). Collaboratively deciding on the client’s goals and the tasks to be accomplished may be more difficult because providers feel they cannot adequately “reach” clients or gather information via videoconferencing needed to agree, align, and form a consensus (Cataldo et al., 2021). Therapeutic presence, an important element for creating psychological and emotional safety (Geller & Porges, 2014), may also be challenging to establish online as providers cannot use their body and non-verbal cues (e.g., vocal tone, leaning forward, gesturing, soft facial features) or read their clients’ facial expressions as easily to communicate support (Geller, 2020).

Alternatively, a virtual space may provide opportunities for enhanced connection and openness with prior research citing greater psychological safety (Stubbings et al., 2015), an “online calming” effect (Reynolds et al., 2013), and a more neutral power balance (Fletcher-Tomenius & Vossler, 2009; Roy & Gillett, 2008) as benefits. Other studies report that therapy via videoconferencing (e.g., teletherapy) facilitates client self-expression and disclosure of difficult feelings and is perceived as less threatening than in-person care (Simpson et al., 2001, 2005, 2021). Providers are also focusing on the development of their “webside manner” (McConnochie, 2019) and enhancing telepresence by ensuring online safety and security (e.g., HIPAA compliance), consistency. and room set-up (e.g., equipment, screen distance, optimal lighting, privacy) and helping clients prepare (e.g., minimize distractions, have tissues and emotion regulation tools, how to transition gently after sessions) (Geller, 2020; Hilty et al., 2019).

Recent empirical evidence has found that therapeutic bonds can be as strong in videoconferencing settings as they are in person. One review of 24 studies found that videoconferencing clients rated their bond (i.e., emotional attachment to the provider) and presence (i.e., the feeling of being “in the moment”) (Geller, 2020) as strongly as those receiving in-person care (Simpson & Reid, 2014). Another review of research on psychotherapy via videoconferencing found high client and provider ratings of alliance that increased over the course of treatment across diagnostic conditions (Thomas et al., 2021). These data are encouraging, yet most individual studies only report average alliance or differences in alliance by environment (in-person vs. videoconferencing) with few evaluating the link between alliance and treatment outcomes, such as subjective well-being or condition-specific symptomatology, especially in video-based interventions (Pihlaja et al., 2018). Although alliance is a key mechanism for improved mental health after therapy (Baier et al., 2020), more research is needed to connect therapeutic alliance as a predictive factor for treatment outcomes, especially in real-world mental health solutions reliant on videoconferencing.

Furthermore, most of the scientific inquiry on therapeutic alliance in digital settings has focused on treatment delivered by therapists or other licensed mental health specialists. Given global gaps in the behavioral health workforce (Health Resources & Services Administration/National Center for Health Workforce Analysis, 2015) and the rising need from the pandemic, mental health services are expanding the criteria for who can provide care. This expansion includes paraprofessionals like certified professional coaches (Clark et al., 2009). Prior research has found that behavioral health coaching is an effective intervention for people with depressive and anxiety symptoms (Montgomery et al., 2010; Sagui-Henson et al., 2021; Theeboom et al., 2014). A recent meta-analysis on in-person coaching found that clients developed high-quality alliance with their coach, which positively influenced their satisfaction and self-efficacy (Graßmann et al., 2020). As digital mental health care evolves to include the provision of more paraprofessional services, we need insight into whether individuals can develop a similar level of alliance with coaches as with licensed therapists.

The purpose of this study was to examine components of therapeutic alliance and the association with well-being outcomes among people with access to employer-sponsored teletherapy and telecoaching during the COVID-19 pandemic. Participants engaged in videoconference sessions with therapists, coaches, or both types of providers, and we evaluated participant-rated components of alliance, subjective well-being and depressive symptoms, and session utilization over time. Our research aims were threefold: (1) evaluate participant ratings of components of therapeutic alliance with providers in a virtual setting, (2) examine changes in subjective well-being and depressive symptoms among participants who screened positive for depressive symptoms at baseline, and (3) explore the association between components of alliance and changes in participants’ well-being.

## Methods

### Design and Participants

We analyzed retrospective de-identified data from global participants who registered for services through a mental health benefits digital platform (Modern Health, Inc., San Francisco, CA). The study time frame was Sept. 29, 20 to Oct. 12, 21. Eligible participants were 18 years or older; received employer-sponsored mental health benefits; had access to a smartphone, tablet, or computer; completed a baseline well-being assessment before their first session; completed a follow-up assessment after their last session at least 7 days after their baseline assessment; completed at least one session with a provider during the study time frame; and provided at least one rating of components of therapeutic alliance for their provider(s). Western Clinical Group IRB reviewed this research and determined it to be exempt from human subjects research.

### Procedures

Participants eligible for the study registered for the platform using a web browser or mobile device. Upon onboarding, participants completed a well-being assessment where they selected topics and symptoms of concern from five areas (emotional, professional, physical, social, and financial health), reported functional impairment related to those areas, indicated their preferred care modality (one-on-one, self-guided, or group care), and completed the World Health Organization-Five (WHO-5) Well-Being Index and other clinical assessments. Based on their assessment results, participants were given a personalized care recommendation of therapy, coaching, and/or use of digital resources. A combination of information from participants’ WHO-5 scores, other clinical assessments, topics of focus, levels of functional impairment, and care modality preferences was used to determine their care recommendation (e.g., a participant was recommended therapy if they selected clinically relevant topics (such as trauma, anxiety, or ADHD), preferred one-on-one care, and reported elevated levels of functional impairment and clinical symptoms). Participants could self-refer into any type of care and could use a mixture of services. There was no prescribed use of the services; participants met with a provider as frequently as they chose to, within the limits of their employer plan with the platform.

### Digital Mental Health Services

People in this study participated in one-on-one care with a provider in the form of teletherapy, telecoaching, or both modalities, which are described below.

#### Teletheraphy

Teletherapy services were offered to participants by licensed clinical therapists who had an advanced degree in clinical psychology or a related field (e.g., Ph.D., PsyD, LCSW, LMFT, or LPC). All therapy visits were conducted virtually through a videoconferencing platform. Therapists were selected for their use of evidence-based practices, such as cognitive behavioral therapy, acceptance and commitment therapy, and dialectical behavior therapy, and internally trained on Modern Health’s proprietary model of care. Teletherapy sessions typically lasted 50 min. The number of therapy sessions a participant attended was dependent on the allotted number of sessions covered by their employer, their therapeutic need, and their level of engagement.

#### Telecoaching

Telecoaching services were offered to participants by professional coaches certified by an International Coaching Federation accredited program and had at least 150 h of coaching experience. Coaching visits were also conducted via a videoconferencing platform. All coaches underwent vetting to ensure their work aligned with evidence-based practices. Although coaches had ample experience and training in core coaching competencies, they also received an additional 6 h of training from Modern Health. This training covered evidence-based techniques (e.g., cognitive behavioral approaches), culturally centered care, how to assess for high-risk situations that may require referring a participant to a therapist or crisis resource, and Modern Health’s proprietary model of care. Telecoaching visits averaged 30 min per session. Coaches worked with clients to recognize self-beliefs and self-limiting behaviors and devise techniques to help them achieve their goals. Like teletherapy offerings, the number of coaching sessions a participant attended depended on the sessions covered by their employer, their therapeutic need, and their level of engagement.

### Measures

#### Demographics

The following demographics were extracted from platform participant accounts: age, sex, and country of residence (dichotomized to “within the US” and “outside the US”).

#### Utilization Type

Participants’ care utilization was classified as: (1) teletherapy only (at least one session), (2) telecoaching only (at least one session), or (3) both teletherapy and telecoaching (at least one session of each type of care). Total session utilization was the total number of teletherapy and/or telecoaching sessions the participant completed during the study period.

#### Components of Therapeutic Alliance

After each session with a provider (therapist or coach), participants were given the option to complete two adapted items from the Working Alliance Inventory (Horvath & Greenberg, 1989; WAI). These items were: “I am confident in [my provider’s] ability to help me” and “[My provider] and I are working on agreed upon goals.” These items represent the bonds and goals components of therapeutic alliance, respectively. The original WAI has response options ranging from 1 (“Never”) to 7 (“Always”). The items used here adapted the response options to a Likert scale from 1 (“Strongly Disagree”) to 5 (“Strongly Agree”). The number of survey items and response options was truncated to facilitate survey completion and encourage higher response rates from participants in a real-world setting. One score was computed for each person by averaging each participant’s response to these two items across sessions (Cronbach *ɑ* = 0.72).

#### Subjective Well-Being and Depressive Symptoms

The WHO-5 (Topp et al., 2015) was used to assess well-being and depressive symptoms before and after care among participants who screened positive for depressive symptoms at baseline (WHO-5 ≤ 28). The WHO-5 is a five-item unidimensional assessment of well-being that asks the participant about their mental well-being over the previous 2 weeks. Items include: “I have felt cheerful and in good spirits” and “I have felt calm and relaxed”. Answers are ranked on a six-point scale 0 (“At no time”) to 5 (“All of the time”), and scores are summed and multiplied by 4, giving a total range of 0–100, with 0 indicating the lowest level of well-being and 100, the highest. The WHO-5 has been shown to exhibit high clinimetric validity as a screening tool for depression (Topp et al., 2015). Clinical recovery from depressive symptoms is defined as a score changing from below to above the clinical cut-off score of 28 (Topp et al., 2015). Clinical improvement in depressive symptoms is defined as an increase of at least 10 points (Bech et al., 2007; Topp et al., 2015).

### Statistical Analysis

We conducted analyses using R version 4.0.4. Our first aim was to evaluate participant ratings of therapeutic alliance components and evaluate whether they differed by demographic group, clinical characteristics, and utilization (provider) type. Given that the scores were strongly positively skewed, non-parametric tests were used to evaluate differences in alliance. To test the association between alliance and age, the Spearman rank-order correlation coefficient was used. To test for differences between the dichotomous variables (sex, participant country (USA or non-USA), and positive screen for depressive symptoms at baseline (WHO-5 score ≤ 28 or > 28)), the Mann–Whitney *U* test was used. Finally, the Kruskal–Wallis test and Dunn post hoc test were used to assess differences in alliance among utilization types.

Our second aim was to examine changes in subjective well-being and depressive symptoms among participants who screened positive for depressive symptoms at baseline (WHO-5 ≤ 28). We focused on this subgroup of participants because their therapeutic and treatment goals were to clinically improve or recover from symptoms, thus anticipating a directional change in scores over time. A paired samples *t*-test was performed to measure changes in well-being from participants’ baseline to follow-up WHO-5 assessment. A between-subjects ANOVA was performed to determine if changes in well-being score (follow-up WHO-5 score minus baseline WHO-5 score) differed by utilization type and used Tukey honest significance post hoc test to explore which groups were significantly different. Percentages were used to determine rates of clinical recovery from and clinical improvement in depressive symptoms (see definitions of these metrics in the “Measures” section).

Our third aim was to evaluate whether the adapted therapeutic alliance score predicted higher well-being at follow-up among participants who screened positive for depressive symptoms at baseline. We again focused on this subgroup because therapeutic alliance is posited to influence improvements in care outcomes. To test this, a linear regression model was constructed that included participant age, sex (dummy coded using female as the reference group), baseline WHO-5 scores, and time in care (number of days between first and last visits) as covariates and examined the change in *R*^*2*^ (incremental variance) from adding the adapted therapeutic alliance score as a predictor of follow-up WHO-5 scores after covariates (Cohen et al., 2003).

We also performed three exploratory analyses using moderated linear regression models to test whether the association between the adapted therapeutic alliance score and follow-up WHO-5 scores differed by the potential moderators: utilization type (dummy coded using teletherapy as the reference group), baseline WHO-5 score, and total session utilization. For each exploratory model predicting follow-up WHO-5 scores, we transformed continuous variables to z-scores (Hayes & Matthes, 2009). In the first model for evaluating each potential moderator, we included the covariates: age, sex (dummy coded using female as the reference group), baseline WHO-5 score (not included as a covariate in the analysis testing baseline WHO-5 as the moderator), and time in care. We also included the adapted therapeutic alliance rating and the hypothesized moderator. We then ran a subsequent model that included the addition of an interaction term (product of z-scored alliance rating and the moderator) and examined the change in *R*^*2*^ (incremental variance) from the previous model containing no interaction term. In the moderated regression testing utilization type (a categorical variable), we evaluated two interaction terms with the adapted therapeutic alliance rating: one for telecoaching only and one for both teletherapy and telecoaching. Teletherapy acted as the reference group. We considered hypothesis tests statistically significant using an *α* level of 0.05.

## Results

### Study Participants

A total of 5985 adults received care and completed assessments during the study period. Among these, 3087 had at least one rating of adapted therapeutic alliance available for analysis. The average age of participants was 35.53 years (*SD* 8.71; range 18–73), 54.1% (1669/3087) identified as female, and 28% (864/3,087) identified as male. Sex was missing from 17.9% (554/3,087) of the sample. Regarding utilization, 18% (555/3,087) of participants utilized care with a therapist, 65% (2,003/3,087) with a professional coach, and 17% (529/3,087) with both types of providers. See Table 1 for the demographic, clinical, and utilization factors in the overall sample and stratified by utilization type.

**Table 1:** Demographic and clinical characteristics among registrants of a digital mental health platform

	Utilization type			
	Teletherapy (*n* = 555)	Telecoaching (*n* = 2003)	Teletherapy and telecoaching (*n* = 529)	Total (*n* = 3087)
Demographic characteristics				
Age, *M (SD)*	35.11 (8.45)	35.80 (8.82)	34.95 (8.51)	35.53 (8.71)
Sex				
Female, *n* (%)	292 (52.6%)	1056 (52.7%)	321 (60.7%)	1669 (54.1%)
Male, *n* (%)	145 (26.1%)	609 (30.4%)	110 (20.8%)	864 (28.0%)
Missing, *n* (%)	118 (21.3%)	338 (16.9%)	98 (18.5%)	554 (17.9%)
Country				
Within USA, *n* (%)	470 (85.1%)	1468 (73.4%)	410 (77.5%)	2348 (76.2%)
Outside USA, *n* (%)	82 (14.9%)	531 (26.6%)	119 (22.5%)	732 (23.8%)
Clinical characteristics				
Components of therapeutic alliance score				
*M* (*SD*)	4.45 (0.76)	4.57 (0.59)	4.54 (0.57)	4.54 (0.62)
Median [min, max]	4.75 [1, 5]	4.83 [1, 5]	4.75 [2, 5]	4.80 [1, 5]
Screened positive for depressive symptoms				
*n* (%)	339 (61.1%)	349 (17.4%)	147 (27.8%)	835 (27.0%)
Follow-up period (days)				
*M* (*SD*)	147.97 (125.95)	129.36 (117.90)	207.60 (125.35)	146.12 (124.04)
Median [min, max]	114 [10, 663]	91 [7, 741]	200 [11, 671]	113 [7, 741]
Total session utilization				
*M* (*SD*)	4.99 (3.48)	2.99 (2.58)	8.65 (5.49)	4.32 (4.01)
Median [min, max]	4 [1, 24]	2 [1, 30]	8 [2, 41]	3 [1, 41]
Time in care (days)				
*M* (*SD*)	50.01 (50.55)	36.66 (52.51)	111.51 (80.72)	51.88 (64.21)
Median [min, max]	36 [0, 303]	16 [0, 337]	99 [1, 351]	28 [0, 351]

The components of therapeutic alliance scores were measured with adapted items representing the bonds and goals components from the Working Alliance Inventory. Follow-Up Period number of days between baseline and follow-up assessments

*M* mean; *SD* standard deviation, *Time in Care* number of days between first and last session

### Components of Therapeutic Alliance and Demographic and Clinical Factors

The median-adapted therapeutic alliance rating was 4.8 and the mean was 4.54 (*SD* = 0.62, range 1–5). Females reported higher median alliance than males (4.88 vs. 4.67, W = 767,724, *P* = 0.005), and the effect size was negligible (*r* = 0.06). The adapted alliance score significantly differed by utilization type (Kruskal–Wallis chi-square = 10.83, *P* = 0.004). A post hoc Dunn test indicated that participants utilizing telecoaching alone (mdn = 4.83) reported a higher median alliance score than those utilizing teletherapy alone (mdn = 4.75, *Z* = 2.70, *P* = 0.02) or both modalities (mdn = 4.75, *Z* = 2.41, *P* = 0.03), and the effect size was negligible (η^2^ = 0.003). There was no association between the adapted alliance score and age (rho = − 0.01, *P* = 0.66). There were no significant differences in the distributions of the median alliance score by country (4.83 within the USA vs. 4.75 outside the USA, *W* = 823,154, *P* = 0.07) or baseline WHO-5 score classification (4.75 WHO-5 ≤ 28 vs. 4.83 WHO-5 > 28, *W* = 909,965, *P* = 0.15).

### Changes in Subjective Well-Being and Depressive Symptoms

Among those screening positive for depressive symptoms at baseline (*n* = 835), participants reported an average WHO-5 increase from baseline to follow-up of 15.42 points (95% CI 14.19–16.65, *P* < 0.001), which constitutes a 75.5% improvement in well-being and large effect size (Cohen *d* = 1.06). Changes in well-being significantly differed by utilization type (*F*(2,832) = 4.76, *P* < 0.001). Participants who utilized both teletherapy and telecoaching (11.32-point increase) reported lower WHO-5 improvements than those who utilized teletherapy only (16.70-point increase, *P* = 0.01) or telecoaching only (15.91-point increase, *P* < 0.03). Well-being changes did not differ between participants who utilized teletherapy only and those who utilized telecoaching only (*P* = 0.84). Of these participants, 58% (485/835) reported clinical recovery from depressive symptoms, and 57.6% (481/835) reported clinical improvement in depressive symptoms based on the WHO-5.

### Components of Therapeutic Alliance and Subjective Well-Being

See Table 2 for regression results for components of therapeutic alliance as a predictor of follow-up well-being among participants who screened positive for depressive symptoms at baseline. In the linear regression model, age (*b* = 0.03, β = 0.01), sex (males, *b* = 0.04, β = 0.002), and time in care (*b* = − 0.01, β = − 0.03) did not significantly predict well-being at follow-up; however, baseline WHO-5 score (*b* = 0.66, *β* = 0.23) predicted significantly higher well-being at follow-up (*R*^*2*^ = 0.05, *P* < 0.001). The adapted therapeutic alliance score (*b* = 2.04, *β* = 0.08) incrementally predicted follow-up well-being (*R*^*2*^ = 0.06, Δ*R*^*2*^ = 0.01, *P* < 0.001). Accordingly, for every one-point increase in the adapted alliance score, follow-up well-being increased by 2.04 points.

**Table 2:** Summary of linear regression analyses for components of therapeutic alliance as a predictor of follow-up well-being scores in participants with elevated depressive symptoms at baseline

	Follow-up well-being						
*Predictors*	*b*	*β*	*SE*	95% CI	*P*	*R*^*2*^	Δ*R*^*2*^
(Intercept)	12.60	0.002	5.83	1.15–24.06	0.03		
Age	0.03	0.01	0.08	− 0.13 to 0.19	0.70		
Sex (male)	0.04	0.002	1.50	− 2.91 to 2.99	0.98		
Time in care	− 0.01	− 0.03	0.01	− 0.03 to 0.01	0.42		
Baseline well-being	0.66	0.23	0.10	0.45 to 0.86	< 0.001	0.05	
Components of Therapeutic Alliance Score	2.04	0.08	0.99	0.09–3.99	0.04	0.06	0.01

n = 675. The components of therapeutic alliance scores were measured with adapted items representing the bonds and goals components from the Working Alliance Inventory. Well-being was measured with the WHO-5. Time in care is the number of days between first and last visits

unstandardized regression coefficient; β standardized regression coefficient, SE standard error, CI confidence interval, R2 variance explained in the outcome, ΔR2 incremental variance explained in the outcome

Exploratory analyses were conducted to test whether the association between components of therapeutic alliance and well-being was moderated by utilization type, baseline WHO-5 score, or total session utilization. Using moderated regression analyses that adjusted for age, sex, baseline WHO-5 score (except for the analysis testing baseline WHO-5 as a moderator), and time in care, we found that the association between components of alliance and well-being did not differ by utilization type (telecoaching: *β* = − 0.001, SE = 0.08, 95% CI − 0.16 to 0.15, *P* = 0.99; both providers: *β* = − 0.001, SE = 0.12, 95% CI − 0.23 to 0.23, *P* = 0.99; *R*^*2*^ = 0.06, Δ*R*^*2*^ = 0.00, *P* < 0.001), baseline WHO-5 score (*β* = − 0.04, SE = 0.04, 95% CI − 0.11 to 0.04, *P* = 0.34; *R*^*2*^ = 0.06, Δ*R*^*2*^ = 0.00, *P* < 0.001), or total session utilization (*β* = 0.03, SE = 0.04, 95% CI − 0.06 to 0.12, *P* = 0.49; *R*^*2*^ = 0.06, Δ*R*^*2*^ = 0.00, *P* < 0.001).

## Discussion

We examined a revised therapeutic alliance scale capturing elements of bonds and goals and their associations with subjective well-being among adults receiving care via videoconferencing as part of a digital mental health platform during the COVID-19 pandemic. We found that participant-rated therapeutic alliance scores were high and did not differ by participant age, country, or baseline well-being; alliance differed by sex and provider type in statistically significant but clinically negligible ways. Females reported slightly higher alliance than males, while those utilizing telecoaching reported slightly higher alliance than those utilizing teletherapy or both services. We also found that the adapted therapeutic alliance score predicted greater well-being at follow-up and that this association did not differ by utilization type, baseline well-being, or total session utilization. Thus, the bonds and goals components of therapeutic alliance appear to be two of many important factors in digital mental health care, and our findings suggest that both telecoaching and teletherapy via videoconferencing can facilitate aspects of therapeutic alliance between clients and providers.

With respect to our first aim, we found that participants were able to form a strong bond and mutually agree upon goals with both coaches and therapists. This may be because the virtual format creates a more neutral and collaborative environment by facilitating care in a familiar setting for the client. It may also provide a greater sense of intimacy and personal control, leading to increased client comfort and investment in the therapy process (Simpson & Reid, 2014). Future research should explore differences in therapeutic alliance between in-person and virtual settings in a real-world context and further examine the mechanisms driving alliance in a videoconferencing environment. Our results also highlight that tailoring care recommendations to people’s clinical needs and personal preferences for a type of provider may be an effective approach to fostering good therapeutic relationships. As telemental health services expand past the subsidence of the COVID-19 pandemic, it will be crucial for digital mental health services to implement ways to systematically measure and track client-provider therapeutic alliance over time to ensure high-quality delivery of care.

This is one of the first studies to compare components of therapeutic alliance between different types of telemental health providers and suggests that professional coaches can form as strong of bonds with their clients as licensed therapists. Telecoaching may lend itself well to developing rapport with clients as coaches focus on helping clients achieve their future goals and address personal development (Grant, 2003), which may in turn result in clients feeling a stronger bond and sense of alignment on goals. This finding also supports global efforts to expand access to mental health care by showing that paraprofessionals can deliver high-quality care. It is important to note that only the bonds and goals components of therapeutic alliance were measured in this study, and we did not have any items from the tasks subscale of the WAI, which captures how relevant, effective, and meaningful a patient finds the tasks of treatment to their goals (Bordin, 1994). Although the therapists and coaches in this study were all trained in and qualified to deliver evidence-based practices (e.g., cognitive behavioral approaches, motivational interviewing), future research should explore the task components to more fully characterize the therapeutic alliance between these two different types of providers. Females also reported slightly higher alliance with the revised scale than males. Although this effect size was small and the difference should be interpreted with caution, prior research shows that females tend to view psychotherapy more favorably than males do (Holzinger et al., 2012), and thus may have entered the relationship with their provider with a greater sense of openness and trust.

With respect to our second aim, well-being scores significantly improved over the course of treatment among participants who screened positive for depressive symptoms at baseline. Throughout the study period, participants’ well-being improved by an average of 15.42 points, representing statistically significant and clinically meaningful increases in well-being. These improvements were similar between participants who utilized teletherapy and those who utilized telecoaching, a promising result given the need for paraprofessionals like professional coaches to help bridge global gaps in behavioral healthcare. Participants who utilized both types of providers reported lower well-being improvements, yet they still reported a more than 10-point WHO-5 increase on average which is indicative of clinically significant change. The observed differences in well-being may be the result of the complexity and uniqueness of this group. Participants utilizing both provider types may have been recommended to see one provider but chose to self-refer to another and may have had different clinical needs than participants who only used one type of provider. We also found that 58% of participants met the threshold for clinical recovery from depressive symptoms, and 57% met the threshold for clinical improvement. These results support previous research on the effectiveness of one-on-one mental health interventions for subjective well-being and depressive symptoms (Sakuraya et al., 2020). The rates of recovery and improvement also align with prior work evaluating the use of videoconferencing to deliver individual psychotherapy (Thomas et al., 2021) and recovery rates for depression in working age adults in stratified stepped care delivery models (Firth et al., 2015).

Regarding our third aim, among participants who screened positive for depressive symptoms at baseline, higher therapeutic alliance scores were associated with greater well-being at follow-up after controlling for baseline well-being and demographic characteristics. This finding reinforces and extends limited prior work that alliance contributes to beneficial treatment outcomes in videoconferencing settings (Pihlaja et al., 2018). Given client preferences and high satisfaction for videoconferencing telehealth (Thomas et al., 2021) and the trend to continue providing telemental health services after the COVID-19 pandemic subsides (Smith et al., 2020), this study contributes encouraging evidence that clients and providers can establish high-quality therapeutic relationships that improve outcomes in virtual care. It also suggests that connecting people with telemental health providers through personalized care recommendations may create beneficial bonds that promote strong outcomes.

Furthermore, one of the barriers to disseminating virtual one-on-one care before the pandemic was low clinician uptake stemming from hesitations that technology inhibits therapeutic processes (Cowan et al., 2019; Guinart et al., 2021). These and other findings should give providers confidence that they can create a collaborative relationship that supports clients’ treatment experience when they are face-to-face but not physically in the same space. Finally, these results also highlight the need to track important contributors to clinical outcomes, including therapeutic alliance and other change mechanisms, in real-world digital mental health interventions to unpack not just whether treatment is effective but why it is effective and how to continually improve those processes to drive outcomes.

### Limitations

Our findings are subject to several limitations. This was an observational study with no comparison group so causal links cannot be drawn between therapeutic alliance and treatment outcomes. We cannot confirm whether improvements in well-being were truly due to alliance or the result of external factors such as the passage of time, societal changes, or a combination of factors. We also do not know how these results would compare to in-person treatment because all the treatment studied here was delivered via videoconferencing. Future research should implement a randomized controlled trial to test differences in alliance and outcomes between provider types and settings or leverage a matched cohort design. Although the longitudinal nature of this study was a strength, there was only outcome data from two timepoints and the adapted alliance score was averaged over treatment sessions. Alliance ratings may have been subject to selection bias wherein the people providing ratings may have had a more positive experience compared to those who did not provide ratings. Although complete data is difficult to capture in real-world settings, future research should aim to utilize data from more timepoints to not miss some of the nuance and variance in alliance and outcomes.

Regarding measurement, the items from the WAI were adapted for use in the digital mental health platform and were not previously validated together. The item battery and response options were reduced to encourage a brief, easy, user-friendly post-session rating experience and increase response rates; as designed, they were intended as a healthcare operations indicator and not a rigorous research metric. The measure of alliance needed to be concise and practical to allow us to have a basic level of clinical insight into session rapport; it was not intended to be a robust measure of alliance. The adapted questions included items from the bonds and goals subscales of the WAI but did not include any items from the task subscale. Future research should investigate the task components of therapeutic alliance because patient-provider agreement on therapeutic tasks may change in the transition from in-person to telemental health services. The revised response options may have also decreased the variance and increased skewness in the components of therapeutic alliance ratings. It was encouraging that internal consistency reliability among the items in this sample was adequate. Because it may not be feasible to ask even the short version of the WAI (12 items) outside of a research setting, more concise, validated, user-friendly measures are needed to track the quality of mental health services in the real world and future research should specifically design measures for this purpose.

Due to historically optional reporting of social identities and demographic factors by employers, we were not able to better characterize our sample to determine the generalizability to a global population. Other important factors that may have affected the outcome in our study were also not assessed, including participant medication usage or outside treatments (either prior to or concurrent with treatment in this study) and attitudes toward or literacy for mental health or telemental health treatment. Finally, while we focused on well-being as our main outcome, we also assessed clinical improvements in and recovery from depressive symptoms using the WHO-5. Although the WHO-5 has strong psychometric properties as both a screening tool and outcome measure (Topp et al., 2015) and may have greater sensitivity and better predictive validity than other depression screeners (Henkel et al., 2003), it was developed as a measure of psychological well-being. Despite these limitations, our findings are strengthened by our capture of real-world effectiveness data on facets of therapeutic alliance and well-being in a large sample of people seeking digital mental health care over an extensive follow-up period.

## Conclusions

Technology-enabled mental health services have the potential to transform how evidence-based care is delivered to be more equitable. The use of videoconferencing to deliver mental health treatment is evolving and will likely play a large role in addressing the needs of people affected by global crises around the world, so ensuring quality is key. This study demonstrated that individuals using teletherapy and telecoaching services through an employer-sponsored mental health benefit reported similarly high ratings of therapeutic alliance with both types of providers, which was a factor that led to effective treatment. This may challenge assumptions that in-person treatment is the only modality through which high-quality relationships can be formed and provokes consideration or reconsideration of the credibility for videoconferencing as a valuable therapeutic medium. It also highlights the importance of tailoring care recommendations to the individual to achieve strong relationships and outcomes. Further research and mental health service evaluation is warranted to ensure that the expansion of digital mental health services is accompanied by the prioritization and centering of client needs and client-provider collaboration.

## Data Availability

Individual de-identified data that underlie the results reported in this manuscript can be shared privately for research purposes upon receipt of a methodologically sound proposal, and whose proposed use of the data from the study related to this article is approved by the authors. To gain access, requesters will need to sign a data access agreement that includes a commitment: (1) to using the data only for research purposes; (2) to not attempt to, or actually, re-identify any individual; (3) to securing the data using appropriate safeguards; and (4) to destroying or returning the data after analyses are completed.
